# Implementation Degree Assessment Sheet for Health Program in Japan by Customizing CFIR: Development and Validation

**DOI:** 10.1186/s43058-022-00270-w

**Published:** 2022-02-19

**Authors:** Reiko Okamoto, Masako Kageyama, Keiko Koide, Miho Tanaka, Yoshiko Yamamoto, Mana Fujioka, Ayami Osuna, Kazuko Saeki, Kazue Hirokane, Fusami Nagano, Shinji Takemura

**Affiliations:** 1 Division of Health Sciences, Osaka University Graduate School of Medicine, Yamadaoka 1-7, Suita-city, Osaka 565-0871 Japan; 2 Former Division of Health Sciences, Osaka University Graduate School of Medicine, Osaka, Japan; 3 Faculty of Nursing, Toyama Prefectural University, Nishi-nagae 2-2-78, Toyama-city, Toyama 930-0975 Japan; 4 School of Nursing, Hyogo University of Health Sciences, Minatojima 1-3-6, Chuo-ku, Kobe-shi, Hyogo 650-8530 Japan; 5 Faculty of Nursing, Fukuyama Heisei University, Kamiiwanari-syoto 117-1, Miyuki-town, Fukuyama-city, Hiroshima 720-0001 Japan; 6 Department of Health Policy and Technology Assessment, National Institute of Public Health, Wako-city-minami 2-3-6, Saitama, 351-0197 Japan

**Keywords:** Implementation science, Assessment tool, Measurement, Public health nurse, Administrative health program, Consolidated Framework for Implementation Research (CFIR)

## Abstract

**Purpose:**

More than 70% of public health nurses in Japan belong to government agencies, and there is a need for further evidence-based capacity development for program implementation. The purpose of this research was to develop an Implementation Degree Assessment Sheet (IDAS) by customizing the Consolidated Framework for Implementation Research (CFIR) to health programs in Japan.

**Methods:**

The draft IDAS (five domains, 31 constructs) created by customizing the CFIR was refined by the researchers and modified through pre-testing. The survey covered full-time public health nurses (PHNs) affiliated to all prefectures and the cities with health centers of Japan. The survey was conducted as an anonymous, self-administered questionnaire survey by mail.

**Results:**

One hundred eighty-five of the 334 institutions (55.4%) agreed to participate in our survey. Of the 966 questionnaire forms distributed, 709 forms (73.4%) were collected, of which 702 responses (72.7%) were valid. No item required consideration of deletion based on the results of item analysis, and our confirmatory factor analysis on model fitness between the five IDAS domains and CFIR showed sufficient fit indices after modification. With regard to reliability, Cronbach’s coefficient alpha, a measure of internal consistency, stayed above 0.8 overall. Our verification of stability with the split-half (odd/even) method resulted in a Spearman–Brown reliability coefficient of 0.95. The correlation coefficient between the IDAS scores and the research utilization competency score, used as an external criterion, was 0.51 (*p*<0.001), supporting the coexistence validity of the criterion-related validity of the IDAS. The significant differences were observed between known-groups, supporting the known-group validity of the IDAS.

**Conclusion:**

This study developed the IDAS and confirmed constant reliability and validity. Hereafter, it is necessary to promote the required capacity development based on the actual degree of implementation in order to use the IDAS for the competency development of public health nurses and related professions to deliver health programs.

**Supplementary Information:**

The online version contains supplementary material available at 10.1186/s43058-022-00270-w.

Contributions to the literature

1. This is meaningful in order to use the developed assessment sheet to comprehensively understand the situation of evidence-based program implementation (EBPI) among administrative health programs and thus clarify how to promote EBPI going forward.
2. The assessment sheet enables: (1) “behavioral assessment” of individual program implementation by having health program practitioners assess their behavior on each construct; and (2) “competency assessment” of program implementation at different levels by calculating the total score and subtotal scores for each domain.

## Background

As health issues have become increasingly diverse and complex, there has been a corresponding increase in administration systems and government measures. The same applies to the activities of public health nurses (PHNs) who are responsible for providing health services to the public. It is therefore critical to implement evidence-based healthcare (EBHC) for using limited financial and human resources effectively and efficiently to resolve health issues [1]. However, actual program implementation is marred by an evidence–practice gap [2]. One way to address this challenge is dissemination and implementation (D&I) science [3], which has developed since the 1990s mainly in the USA. In Japan, insights gained from D&I could be used to promote evidence-based program implementation (EBPI).

PHNs are the primary drivers of health programs in Japan. In order to maintain and promote the health of individuals and the whole community and to prevent diseases, public health nurses develop health activities based on community diagnosis and according to community characteristics [4]. Under the Japanese PHN qualification system, which was launched in 1941, 73.9% of PHNs (39,117 or 30.9 PHNs per 100,000 persons) are affiliated with government agencies [5]. The direction of their activities is mainly defined as the “implementation of a PDCA cycle” under the National Guidance on PHN Activities [4], thus requiring EBPI. This direction is described as “capacity of program and policy development required to find solutions to local health issues” under the National Guidelines on PHN Training [6], whereas the career ladder pertaining to expertise after graduation includes “program and policy development” capacity to solve local health issues, as one of the six areas of activities to be implemented by PHNs [7], which means that EBPI is an essential activity and capacity to be acquired by PHNs.

However, a PHN survey (*n*=604) [8] that measured research utilization competency (RUC) with a two-factor, ten-item scale containing five evidence-based steps: (1) Ask, (2) Acquire, (3) Appraise, (4) Apply, and (5) Assess [9], showed low scores overall and for two skill subsets in particular—“To examine the search results to solve on-site problems (that contain the contents of step 1-2)” and “To appraise, apply, and evaluate the search results (that contain the contents of step 3-5)”—namely 58.7, 63.7, and 53.7 out of 100 points, respectively. These results indicate that, despite the widespread recognition of the importance of evidence among PHNs, there are many obstacles to actually using it.

The whole process of EBPI incorporates many organizational factors in addition to the five processes mentioned above. In order to promote EBPI by PHNs in future, it is necessary to grasp the situation not only of RUC but also of the evidence–practice gap in the whole EBPI process and consider the appropriate direction of problem-solving based on the results. However, the situation of EBPI in Japan is still not known, not only for public health nurses, as no comprehensive assessment sheet has been developed on this subject.

Accordingly, our research team considered the possibility of using an existing framework for D&I research as an assessment sheet. After analyzing 111 studies using various D&I conceptual frameworks, a review by Mazzucca et al. [10] reported that the Consolidated Framework for Implementation Research (CFIR) [11] and the RE-AIM Model (which has five factors: reach, efficacy, adoption, implementation, and maintenance) [12] were the most commonly used frameworks, each used in 16 of the 111 studies. After examining the most popular models, we found that CFIR and RE-AIM had an advantage in the comprehensiveness of “implementation” ranging from the adoption to introduction of evidence, and of the evaluation of interventions, respectively. Since the purpose of this study was to comprehensively understand the actual status of EBPI among public health nurses, CFIR, which emphasizes the process of adopting evidence-based interventions, was chosen as the most appropriate for this study. Since CFIR includes “1. Intervention Source, 2. Evidence Strength & Quality, 3. Relative Advantage, 4. Adaptability”, we believe that CFIR will contribute to strengthening the research utilization competency of PHNs. CFIR is a framework developed by integrating about 20 theories and models from psychology, sociology, organizational change theory, etc. The number of citations in PubMed exceeds 2600 as of July 2021, and it is being used in many countries.

### Purpose of research

The purpose of this research was to develop an Implementation Degree Assessment Sheet for Health Program (IDAS) by customizing CFIR. The study design was the development and validation of the items and the scale for the implementation of health programs.

This is important in order to use the sheet to comprehensively understand the situation of EBPI among Japanese PHNs and thus clarify how to develop the capacity of public health nurses to promote EBPI going forward.

The term “program” was defined as “any program, activity, or other engagement with the targets of public health (individual, family, population, organization, local or other community), organized by you or members of your department.”

## Methods (Table 1)

The development and validation of the IDAS followed the three phases of Boateng et al. [13].

**Table 1:** Procedure of IDAS development and validation

Phase	Step	Method	Purpose/adoption criteria
Phase 1: Item development	Step 1: Identification of domain and item	Choose an existing framework that has been validated.	Because CFIR (five domains, 38 constructs) is a meta-theoretical framework that integrates about 20 theories and models and has already been used and validated in many studies (over 2600 citations).
	Step 2: Content validity	Forward translation and reconciliation: 4 individuals translated, consulted, and integrated. Modify the contents to fit the context of Japanese health programs: 7 experts consulted.	To ensure the quality of the translation by adhering to the forward translation procedure by multiple people. To ensure the quality of item selection and regeneration by Japanese experts with experience of health program implementation.
Phase 2: Scale development	Step 3: Pre-testing	Pre-test with the target group	To ensure the questions and answers are meaningful. Adopt those with at least 70% agreement for each question of "easy to understand" and "think it is important”.
	Step 4: Survey administration and sample size	National survey of the target population	To ensure the quality of reliability and validity by calculating a sufficient sample size for the survey and distributing it evenly throughout the country.
	Step 5: Item analysis	Ceiling/floor effect	To examine whether there is a scale attenuation effect depending on the degree of asymmetrically of the frequency distribution. Items with mean + 1SD < 5 or mean − 1SD > 0 are deleted.
		Item-total correlation analysis	To check if any item in the set of tests is inconsistent with the averaged behavior of the others, and thus can be discarded. Item-total correlation coefficient ≥ 0.5
Phase 3: Scale evaluation	Step 6: Tests of reliability	Cronbach’s coefficient alpha	To measure the internal consistency of IDAS. Adopted at 0.8 or higher.
		Split-half correlations (odd/even) method	To check the verification of stability of IDAS. Adopted at the Spearman–Brown reliability coefficient 0.8 or higher.
	Step 7: Tests of validity	Construct validity	To verify the model fitness of construct validity with a confirmatory factor analysis. Check the goodness-of-fit index for fit in the same five domains as CFIR GFI, CFI ≥ 0.9 RMSEA < 0.08
		Criterion validity	To estimate the extent to which a test correlates with an established standard of comparison (RUC). Correlation coefficient ≥ 0.5
		Differentiation by “Known Groups”	To examine if the concept measured behaves as expected in relation to “known groups”. (Four groups depending on the years of experience and whether the respondent was a supervising PHN or not) Significant difference between the groups (*P* < 0.05)

### Phase 1: Item development (Table 1)

#### Step 1: Identification of domain and item

We used CFIR which had the five major domains and 38 constructs [14] as the framework for the IDAS, and the items were determined by discussing with the co-researchers of the lead university whether the contents could be used to assess the degree of health program implementation based on EBPI in Japan. CFIR was originally developed by integrating numerous existing models [11] and has been used across a wide range of studies for more than a decade [15]. Regarding the use of the CFIR, we confirmed that it was open and free to the developer, Damschroder.

#### Step 2: Content validity

Content validity was ensured through weekly consultations among the co-researchers over two months. Specifically, each of the four members read CFIR webpages and translated the CFIR constructs and guides into Japanese. We collated the translated materials and generated IDAS constructs by closely examining whether each construct is relevant to specific, hypothetical cases of health programs in Japan. We used taxonomy verbs for expression as they may be used for assessing behavior. These items were subsequently modified to fit the context of health programs in Japan by seven expert members with experience in health program implementation and qualitative research. For example, we adopted for number 11 non-competitive content in the program development of Japanese administrative PHNs because of the characteristic of emphasizing “Horizontal Development of Pioneering and Superior Practice” rather than “Peer Pressure.”

Consequently, all five domains were retained, while the constructs were ultimately reduced to 31 after consolidation and reorganization. As detailed in the footnotes to Table 3, the two CFIR items were reorganized to fit the Japanese context (IDAS; No. 7, 11), and the 11 CFIR items were integrated into the five IDAS items.

In order to make the IDAS available internationally in the future, the English version of the IDAS was prepared in accordance with the method of Wild et al. [16] by following the steps of (1) Forward Translation, (2) Back Translation, and (3) Backward Translation Review.

### Phase 2: Scale development (Table 1)

#### Step 3: Pre-testing

In order to evaluate content validity, a pre-test of a target group was conducted after participants attended a training session on program implementation. The training session lasted 90 min and consisted of a PowerPoint lecture introducing the importance of EBPI and IDAS for public health nurses, and group work to discuss their own level of implementation. There were two questions in the pre-test, whether each item was easy to understand and whether the participants recognized its importance. We selected the constructs receiving at least 70% approval regarding the two questions.

#### Step 4: Survey administration and sample size

The survey covered PHNs affiliated to 334 government offices and health centers of all prefectures and the cities with health centers. This method was chosen because there are two types of health centers run by government agencies in Japan: prefectural and municipal. We selected health centers by stratified random sampling and requested six PHNs per center to participate. Assuming that 50% of the institutions and PHNs would participate, we expected data collection from some 500 respondents. Thus, the survey was designed to secure a sufficient sample size of 400, with a population of 40,000, confidence level of 95%, error margin of 5%, and response rate of 0.5.

The survey was conducted as an anonymous, self-administered questionnaire survey by mail to each head PHN. The head PHNs were asked to minimize bias in terms of job title, years of experience, and function in distributing the questionnaire form. We asked the participants to return the questionnaire form in a separate envelope provided by the researcher, which could be returned unsigned, to ensure that the responses were voluntary. The survey was conducted in January 2020.

The survey pertained to basic attributes and the draft IDAS (five domains and 31 constructs of CFIR customized to health programs in Japan).

The question asked: “Each of the following constructs is a statement concerning behavior in adopting/implementing a new program. Does the statement apply to your work behavior?” The participants were asked to assess their behavior on a scale of 0 to 5, ranging from “0: Does not apply at all” to “5: Always applies” (with the total score falling between 0 and 155; refer to Table 3 for the items).

#### Step 5: Item analysis

As item analysis, we conducted a ceiling/floor effect analysis [17] and an item-total correlation analysis. The adoption criteria are shown in Table 1.

### Phase 3: Scale evaluation (Table 1)

#### Step 6: Tests of reliability

Regarding reliability, we checked internal consistency with Cronbach’s coefficient alpha, and stability with the split-half correlations (odd/even) method.

#### Step 7: Tests of Validity

Validity was verified in terms of construct validity, criterion-related validity, and known groups validity (four groups depending on the years of experience and whether the respondent was a supervising PHN or not). Since the IDAS comprises CFIR’s five domains, we verified the model fitness of construct validity with a confirmatory factor analysis.

The external criterion for criterion validity was the Research Utilization Competence Scale (RUC) score, which consists of 10 items (ranging from “Does not apply at all: 1” to “Most often applies: 6”; 10–60 points) and has been validated for reliability and validity [8].

As statistical software, we used IBM SPSS ver.27 (containing Advanced Statistics, Regression, and Amos) with a significance level of 5%.

### Ethical considerations

We explained the ethical considerations in a document sent with the questionnaire form. The research program was implemented with the approval of the Ethical Review Board, Osaka University Hospital (Approval No. 19285 dated November 5, 2019). The document also explained the voluntary nature of participation in the survey, protection of personal information, data processing policy, and the publication of research findings, among others. The respondents gave consent to participation in the survey by ticking the appropriate box in the document and returning it.

## Results

### Phase 1: Item development

#### Step 1: Identification of domain and item

The draft IDAS, created following a series of consultations, contained all of the five domains of CFIR, as well as 31 constructs customized to heath programs in Japan (see constructs in Table 3). Although the original CFIR had 38 constructs [14], we customized them by narrowing and consolidating the meanings of those constructs which do not fit the scale of programs executed by PHNs or the structure of administrative organizations in Japan.

#### Step 2: Content validity

Thus, the definition of Construct I-G “Design Quality & Packaging” was narrowed down to mean “Material Quality Control.” Construct II-C “Peer Pressure,” which literally refers to pressure from companies operating in the same industry or competitors, was changed to “Horizontal Development (of Pioneering and Superior Practices)” to include positive impact. Construct III-D “Implementation Climate (1. Tension for Change, 2. Compatibility, 3. Relative Priority)” was modified to “Fostering Change Acceptance Climate” to put more weight on infrastructure development. As the scope of our survey is limited to program implementation by administrative organs, Construct A “Structural Characteristics” was narrowly defined in the spirit of Sub-constructs D5 and D4 as “17. Goals Setting and Accountability” and “18. Confirmation of Higher Goals/Incentives.” The five sub-constructs of Construct “V-B. Engaging” were modified into expressions and statements intelligible to Japanese PHNs, focusing on important factors of program implementation by administrative organs, namely internal human resource development, external partnerships, and program participants. Thus, Sub-constructs 1 “Opinion Leaders,” 2 “Formally Appointed Internal Implementation Leaders” and 3 “Champions” under Construct B “Engaging” were integrated into a construct called “Engaging: Internal Implementation Key Persons.” We also consolidated Sub-constructs B4 and B5 into “Engaging: External Change Agents/Key Stakeholders.”

### Phase 2: Scale development

#### Step 3: Pre-testing

The number of participants in the training session was 7, and their attributes included an average of 7.7 years of experience as PHNs working for an administrative organ (range 2–15). The respondents had no difficulty understanding any of the statements. At least five PHNs (71.4%) recognized all but one construct as important, with the exception of “Trialability,” pointing to content validity. We revised the expression of “Trialability” in Japanese by adding the word “confirmation.”

#### Step 4: Survey administration and sample size (Table 2)

One hundred eighty-five of the 334 institutions (55.4%) agreed to participate in our survey. Of the 966 questionnaire forms distributed, 709 forms (73.4%) were collected, of which 702 responses (72.7%) were valid.

**Table 2:** Basic attributes of participants * N*=702

Attributes		Mean ± SD	*n*	%
Gender	Female		672	95.7
	Male		30	4.3
Years of experience as a PHN		22.6 ± 11.6		
	1–5		87	12.4
	6–15		120	17.1
	16–25		132	18.8
	26 or more years		363	51.7
Affiliated administrative agency^a^	Prefectural government		175	24.9
	Health center		527	75.1
Supervising public health nurse	No		561	79.9
	Yes		141	20.1
Job title	Team leader/member		279	39.7
	Section head or higher^b^		423	60.3

^a^Includes prefectural government or city with health centers

^b^Includes section head, assistant manager, manager, director or higher job title, or supervising PHN

The attributes of the participants (Table 2) indicated that women accounted for an overwhelming majority (95.7%), over half of whom (51.7%) had experience of 26 years or over. By affiliation, 24.9% were working for a prefectural government, and 75.1% at a health center. By job title, about 20% were supervising PHNs and 60% had a position equivalent to section head or higher.

#### Step 5: Item analysis (Table 3)

In our item analysis, any ceiling/floor effects were found, as there were no values with a mean +1SD less than 5 and a mean −1SD greater than 0. The item-total correlation analysis found no extremely low correlation coefficient of less than 0.5 between item and total scores, ranging between 0.557 and 0.717.

**Table 3:** Item analysis

Domain	Construct		Short description	Mean ± SD	Item-total correlation analysis^a^	
					Correlation coefficient	*p* value
I Intervention characteristics	1	Intervention source	Know how the intervention is developed.	3.9 ± 0.8	0.58	< 0.001
	2	Evidence strength and quality	Know the extent to which the intervention is evidence-based.	3.6 ± 0.9	0.59	< 0.001
	3	Relative advantage	Know the advantage of the intervention versus an existing project.	3.7 ± 0.9	0.62	< 0.001
	4	Adaptability	Clarify how the intervention can be modified or adjusted to meet local needs.	3.9 ± 0.8	0.70	< 0.001
	5	Trialability	Introduce a pilot phase before full implementation.	2.9 ± 1.1	0.58	< 0.001
	6	Complexity	Clarify conditions for implementation (including procedure, scope, and period).	3.9 ± 0.8	0.72	< 0.001
	7	Material quality control	Prepare teaching aids and materials to guarantee the quality of the intervention.	3.6 ± 0.8	0.60	< 0.001
	8	Cost	Qualify the costs associated with implementing the intervention by expense item.	3.9 ± 0.9	0.64	< 0.001
II Outer setting	9	Patient needs and resources	Clarify the need to implement a new intervention in response to the trend of health issues.	4.0 ± 0.8	0.66	< 0.001
	10	Cosmopolitanism	Identify interventions in other regions or by other organizations and exchange views and information thereon.	3.7 ± 0.8	0.60	< 0.001
	11	Horizontal development of Pioneering and superior practice	Identify advanced good practices and their implementation in other regions or by other organizations.	3.7 ± 0.8	0.61	< 0.001
	12	External policy and incentives	Identify and utilize trends in central and prefectural government policies in a timely manner.	3.7 ± 0.9	0.69	< 0.001
III Inner setting	13	Readiness for implementation: available resources	Identify and prepare the space and equipment for implementing the intervention.	4.0 ± 0.8	0.56	< 0.001
	14	Networks and communications	Hold meetings to consult on implementation and secure communication tools such as e-mail and telephone.	4.0 ± 0.7	0.60	< 0.001
	15	Culture	Take into account the impact of organizational culture (including norms, values, and characteristics).	3.6 ± 0.9	0.62	< 0.001
	16	Fostering change acceptance climate	Ensure that the organization recognizes and accepts the priority and importance of the new intervention.	3.8 ± 0.8	0.70	< 0.001
	17	Goals setting and accountability	Ensure that the organization sets and publishes the goals to be achieved by the intervention.	3.5 ± 0.9	0.63	< 0.001
	18	Confirmation of higher goals/incentives	Confirm consistency with higher goals (such as comprehensive plan or basic guidelines).	3.9 ± 0.8	0.70	< 0.001
	19	Implementation climate: learning climate	Ensure that the organization develops a culture and system to gain knowledge and skills required for the intervention.	3.5 ± 0.9	0.71	< 0.001
	20	Readiness for implementation: leadership engagement	As a leader, explain the details of the intervention to the team members and support their roles.	3.7 ± 0.9	0.71	< 0.001
	21	Readiness for implementation: access to knowledge and information	Develop an environment for the intervention team members to improve their competencies (opportunities for training and provision of teaching aids, etc.).	3.6 ± 0.9	0.72	< 0.001
IV Characteristics of individuals	22	Knowledge and beliefs about the intervention	Have the knowledge, skills and belief required for one's own intervention.	3.9 ± 0.7	0.68	< 0.001
	23	Self-efficacy	Have belief in one's own capabilities/a sense of self-sufficiency in implementing the intervention.	3.7 ± 0.8	0.64	< 0.001
	24	Individual stage of change	Be prepared to implement each phase of the intervention on one's own (knowledge/persuasion/decision/execution/confirmation).	3.9 ± 0.7	0.68	< 0.001
	25	Individual identification with organization	Take pride in displaying one's ability at the workplace.	3.5 ± 0.9	0.62	< 0.001
V Process	26	Planning	Ensure that the organization rigorously develops a feasible execution plan.	3.8 ± 0.8	0.67	< 0.001
	27	Engaging: internal implementation key persons	Place key persons in supervising/directing positions and the execution team.	3.4 ± 1.0	0.61	< 0.001
	28	Engaging: external change agents/key stakeholders	Partner/collaborate as necessary with relevant external parties and organizations.	4.0 ± 0.8	0.68	< 0.001
	29	Engaging: intervention participants	Recruit intervention participants via multiple publication media/channels.	3.6 ± 0.9	0.57	< 0.001
	30	Executing	Carry out or accomplish the intervention according to plan.	4.0 ± 0.7	0.67	< 0.001
	31	Reflecting and evaluating	Regularly review the progress of execution for evaluation and improvement.	4.0 ± 0.8	0.70	< 0.001

CFIR original contracts customized for health programs in Japan:

7←Design Quality & Packaging, 11←Peer Pressure, 16←D1·2·3: Implementation Climate; Relative Priority/Compatibility/Tension for Change, 17/18←A: Structural Characteristics/D4·5: Implementation Climate: Organizational Incentives & Rewards/Goals and Feedback, 27←B1·2·3: Engaging; Opinion Leaders/Formally Appointed Internal Implementation Leaders/Champions, 28←B4·5: Engaging; External Change Agents/Key Stakeholders

^a^Correlation analysis

### Phase 3: Scale evaluation

#### Step 6: Tests of reliability

With regard to reliability, Cronbach’s coefficient alpha, a measure of internal consistency, stayed above 0.8 overall and for each of Domains I–V (0.95, 0.86, 0.83, 0.88, 0.89, and 0.86, respectively). Our verification of stability with the split-half (odd/even) method resulted in a Spearman–Brown reliability coefficient of 0.95.

#### Step 7: Tests of validity

We used confirmatory factor analysis to test whether the five domains of the IDAS fit the five-factor structure. The results initially showed inadequate fit indices of GFI: 0.78, CFI: 0.84, and RMSEA: 0.08. We added covariance to the 15 locations among the error variables where the value of the adjusted index was greater than 30. In doing so, we checked the content of the paired items and limited the covariance to those items for which a relevant context was identified. Then the values of fitness indexes showed improvement, although the GFI was slightly lower than the criterion of 0.9, the DFI was higher than 0.9 and the RMSEA was lower than 0.08, it reached acceptable levels of GFI: 0.87, CFI: 0.92, and RMSEA: 0.06.

The IDAS score and the research utilization competency score, used as an external criterion, showed a correlation coefficient of 0.51 (*p*<0.001). The result of the known-group method analysis (Table 4) showed significant differences in the IDAS score among the four groups of years of experience, as well as between the two job title groups (supervising PHN or not) (*p*<0.001). Multiple comparisons indicated significant score differences between Group 1 (1–5 years) on the one hand and Groups 3 (16–25 years) and 4 (≥26 years) on the other, as well as between Group 2 (6–15 years) and Group 4 (≥26 years) (*p*<0.01).

**Table 4:** Relationships between known-groups *N*=702

Known-groups		*n*	Mean ± SD	*p* value^a^	Multiple comparison^b^	
Years of experience as a PHN	① 1–5	87	107.1 ± 20.4	< 0.001	①<③	0.003
	② 6–15	120	111.6 ± 17.4		①<④	<0.001
	③ 16–25	132	115.9 ± 14.2		②<④	<0.001
	④ 26–	363	119.1 ± 15.4			
Supervising public health nurse	No	141	114.2 ± 16.7	< 0.001		
	Yes	561	121.9 ± 15.8			

^a^One-way ANOVA

^b^Games-Howell

## Discussion

The reliability and validity of the IDAS may be described in line with the three phases [13].

Phase 1: Item Development. We believe that the confirmability of “Identification of Domain and Item Generation” was ensured by using CFIR, a framework that has been used and developed over a decade in various countries. Content validity was also ensured by ongoing evaluation and modification by expert co-researchers and by modification based on the results of the pre-test.

Phase 2: Scale Development. Our survey obtained 702 valid responses. Along with the smallest attribution unit of 87 samples used for the analysis, we secured an adequate size of samples representing the population. No ceiling or floor effect was found, no item required consideration of deletion in the item-total correlation analysis, either.

Phase 3: Scale Evaluation. Internal consistency and stability were verified to warrant the reliability of the IDAS. The correlation between the IDAS and external criteria and the expected score differences among “known groups” confirmed the criterion validity (concurrent validity and known-group validity). Construct validity was also confirmed as our confirmatory factor analysis attested to the model fitness of the five IDAS domains.

From these results, we developed the IDAS, composed of five domains and 31 constructs with certain levels of verified reliability and validity.

Next, the following is a discussion of how the IDAS can be used to develop the capacity of public health nurses to promote EBPI. However, as a prerequisite, it should be noted that not all of the constructs need to be adhered to in actual work. The reason for this is that within each organization, there are different areas of responsibility depending on the division of work and position.

First, it may be used to assess individual behavior in program implementation by checking each of the constructs. For example: (1) the person responsible for implementing a health program may use the IDAS for regular self-assessment to identify (i) where the requirements have been met, (ii) where improvements have been observed, and (iii) where problems exist, thus facilitating his/her skill upgrading going forward; or (2) at the workplace level, individual PHNs may use the result of their self-assessment (i) to exchange information on their current status by construct and (ii) to share best and worst practices, thus facilitating individual skill upgrading and improvement. In the researches that used CFIR for program evaluation [18, 19], the status of each construct was compared for each facility to identify constructs that strongly differentiated whether the implementation was effective or not, so that improvements could be considered from the analysis. If we replace this method of utilization with the IDAS, we believe that the IDAS can be used as a tool to identify constructs that require capacity building at the individual level and apply it to continuous self-improvement.

Second, the total score and the scores for individual domains may be used for “competency assessment” in program implementation at the workplace. Concrete examples may include (1) regularly aggregating the self-assessment results of all members at the workplace level to identify any domain in which the workplace may be experiencing problems in competency, to serve as an input to competence development planning; or (2) identifying historical trends to inform quality improvement and future planning at the workplace level. Researches using CFIR have analyzed community-based programs for promoting and obstructing factors within the system or organization [20–22]. This suggests that if the IDAS can be used at the workplace level for a specific program, or at the team level, including related organizations, we believe that the IDAS can be used to identify strengths and challenges of the system in a community or organization on the basis of the aggregated scores for each domain, and to find guidelines for capacity building that should be undertaken by the entire team.

Finally, each domain or construct of the IDAS customized for health programs in Japan might serve as a framework for formative evaluation of program implementation in Japan, on the grounds that the original CFIR contains cumulative evidence [10, 15, 23] that can be used for formative evaluation of programs and activities. In doing so, interpreting the high and low scores requires an open discussion of whether or not it was a necessary construct for evaluating the degree of implementation of the program. We believe that continuing these researches, accumulating the results, and deepening the dialogue will contribute to the implementation science.

## Limitations

A methodological limitation of this study is the issue of the representativeness of the population. Although a sufficient sample size was obtained, the percentage of the group with experience of 26 years or more was more than 20% higher than actual, and the percentage of the group with 6–15 years and 16–25 years was more than 10% lower. The need for further investigation and consideration of this effect is an issue for the future. Another limitation of this study is that the fitness index GFI did not meet the criterion, which we would like to discuss in future research. This research was intended to develop an assessment sheet to understand comprehensively the situation of EBPI among Japanese PHNs and was limited to the preparation of the IDAS, comprising five domains and 31 constructs. Future challenges include developing guidelines to promote EBPI and closing the evidence–practice gap by enhancing the elements of the IDAS through ongoing qualitative research that may serve as behavioral indicators. In addition, because of the limitations of IDAS, which is composed of items that fit the Japanese context, the transferability of IDAS for international use needs to be examined in the future.

## Conclusions—development and validation of the IDAS

The IDAS (five domains, 31 constructs) was developed by customizing the five domains of the CFIR and the constructs included in it to assess the degree of implementation of health programs in Japan, and by going through three phases of a rigorously set procedure to ensure reliability and validity.

In the future, it is hoped that the IDAS will be used for developing the capacity of public health nurses and related professions, and for evaluating the implementation of programs. It is necessary to continue to accumulate research results to enhance the content of the IDAS as behavioral indicators, and to examine the possibility of its use in other countries.

## Supplementary Information

**Additional file 1.**

## Data Availability

The datasets analyzed during the current study are not publicly available due to needing further analysis but are available from the corresponding author on reasonable request.
